# Salutatory

**Published:** 1853-07

**Authors:** 


### EDITORIAL DEPARTMENT.

*Salutatory.*—The junior editor, in taking his place in the chair editorial, derives confidence in his new position from the character of his senior, and from the kindness with which his previous lucubrations have been received. In thus entering upon a new and untried vocation, he has been troubled by some qualms of conscience, and some hesitating doubts as to his ability, which time must solve. He makes no promises, and does not anticipate a wide influence in a field already filled by abler men.

Only this—in devotion to legitimate medicine, and in an earnest desire to make himself useful to his profession and to the readers of the Journal; he will not fail. S. B. H.

